# How Does Pollen Chemistry Impact Development and Feeding Behaviour of Polylectic Bees?

**DOI:** 10.1371/journal.pone.0086209

**Published:** 2014-01-21

**Authors:** Maryse Vanderplanck, Romain Moerman, Pierre Rasmont, Georges Lognay, Bernard Wathelet, Ruddy Wattiez, Denis Michez

**Affiliations:** 1 Research Institute of Biosciences, Laboratory of Zoology, University of Mons, Mons, Belgium; 2 Unit of Analysis Quality and Risk, Laboratory of Analytical Chemistry, Gembloux Agro-Bio Tech, University of Liege, Gembloux, Belgium; 3 Unit of Biological and Industrial Chemistry, Gembloux Agro-Bio Tech, University of Liège, Gembloux, Belgium; 4 Research Institute of Biosciences, Department of Proteomic and Protein Biochemistry, University of Mons, Mons, Belgium; 5 Evolutionary Biology and Ecology, Université Libre de Bruxelles, Brussels, Belgium; University of Sussex, United Kingdom

## Abstract

Larvae and imagos of bees rely exclusively on floral rewards as a food source but host-plant range can vary greatly among bee species. While oligolectic species forage on pollen from a single family of host plants, polylectic bees, such as bumblebees, collect pollen from many families of plants. These polylectic species contend with interspecific variability in essential nutrients of their host-plants but we have only a limited understanding of the way in which chemicals and chemical combinations influence bee development and feeding behaviour. In this paper, we investigated five different pollen diets (*Calluna vulgaris*, *Cistus* sp., *Cytisus scoparius*, *Salix caprea* and *Sorbus aucuparia*) to determine how their chemical content affected bumblebee colony development and pollen/syrup collection. Three compounds were used to characterise pollen content: polypeptides, amino acids and sterols. Several parameters were used to determine the impact of diet on micro-colonies: (i) Number and weight of larvae (total and mean weight of larvae), (ii) weight of pollen collected, (iii) pollen efficacy (total weight of larvae divided by weight of the pollen collected) and (iv) syrup collection. Our results show that pollen collection is similar regardless of chemical variation in pollen diet while syrup collection is variable. Micro-colonies fed on *S. aucuparia* and *C. scoparius* pollen produced larger larvae (i.e. better mates and winter survivors) and fed less on nectar compared to the other diets. Pollen from both of these species contains 24-methylenecholesterol and high concentrations of polypeptides/total amino acids. This pollen nutritional “theme” seems therefore to promote worker reproduction in *B. terrestris* micro-colonies and could be linked to high fitness for queenright colonies. As workers are able to selectively forage on pollen of high chemical quality, plants may be evolutionarily selected for their pollen content, which might attract and increase the degree of fidelity of generalist pollinators, such as bumblebees.

## Introduction

Pollen is one of the prime nutrient resources used for adult and larval development of bees [1]–[3]. Major components of pollen include lipids, carbohydrates, proteins, amino acids, vitamins, carotenoids and flavonoids [4]–[7]. Pollen chemical content is species-dependant but closely related floral species could display similar composition [7]–[12]. Interspecific variability in essential nutrients (e.g. proteins, sterols and carbohydrates) may be a constraint for bee development and species permanence at least for polylectic bees that mix pollen resources (i.e. constraint hypothesis developed by [13]–[15]). Previous studies on the generalist species *Apis mellifera*, *Bombus terrestris*, *Osmia bicornis* and *Osmia cornuta* confirmed that some pollen diets are inadequate for bee development [14]–[19]. Assessment of pollen quality based on chemical composition may thus be a factor driving foraging behaviour in polylectic species [11], [20]. Previous empirical studies, however, did not analyze a significant range of pollen nutrients and did not evaluate the impact of the presence of these nutrients on global feeding behaviour (pollen and nectar collection). The effects of pollen quality on the development and behaviour of polylectic bees thus remain largely unknown.

Pollen nutritional value is currently evaluated by its crude protein content (i.e. evaluated from nitrogen content) as protein level is crucial for reproduction, growth, immunocompetence and longevity of bees and insects in general [5], [11], [17], [21]–[26]. However, the “protein” value does not distinguish different molecules like polypeptides, free amino acids or essential amino acids that display diverse physiological functions [27], [28]. On the one hand, “polypeptides” (molecular weight >10000 Da) can enhance immune functions in insects [24], [25] and play other functional roles in insect diets such as binding fats, binding flavours (i.e. polypeptides have little flavor of their own, but influence flavor perception via binding and/or adsorption of flavor compounds) and storage [29]. They may act as emulsifiers and may give the diet greater elasticity or other texture features that may be either desirable or detrimental. Moreover polypeptides include enzymes that may impact the nutritional value of diets, including the degradation of nutrients such as lipids and proteins or the formation of insoluble or indigestible complexes [29]. On the other hand, amino acids include free amino acids and protein-bound amino acids. Whereas free amino acids can be modified in non-protein analogs which are toxic for bees [29], protein-bound amino acids constitute the usable part of the total amino acids in pollen (including the essential amino acids; [29]). Like many animals, bees need to assimilate certain essential amino acids from their food [16], [30]. The ideal composition of essential pollen amino acids was determined for the honeybee, *Apis mellifera*, by De Groot [16]. These amino acids are the same as those needed by other animal taxa [30]. Thus, it can be assumed that bees do not vary significantly in their nutritional requirements concerning relative amino acid composition.

Growing evidence suggests that high protein content in pollen may be detected by polylectic species, given that some species, like bumblebees, forage preferentially on pollen with high protein content [11], [20]. Furthermore, it seems likely that amino acid composition has a greater influence on the amount of pollen required by bees than crude protein content [31].

Another contributing factor to host plant suitability may be sterols [32], which play an essential role in hormone synthesis, gene expression and cell membrane function [27], [32]. Not all insects can synthesize these compounds that must thus be ingested. As nectar contains extremely low levels of sterols, bees must assimilate these compounds from pollen [33]. More than a hundred different sterols have been identified in plants [34]. These phytosterols (i.e. dominant plant sterols) have generally additional carbon(s) compared to cholesterol, the most used and usually sufficient sterol for insect requirements [32]. Some insects are able to dealkylate phytosterols into cholesterol but this ability is lost in the more derived members of Hymenoptera like bees [32]. As far as known, bees use alternatively synthesis of a particular moulting hormone from phytosterol (C_28_ Makisterone A or C_29_ Makisterone C in place of C_27_ Ecdysone synthetized from C_27_ cholesterol) [35]. It seems likely that dietary sterol requirements affect feeding behaviour of generalist bees [32]. This hypothesis was partly corroborated by studies showing that the generalist honeybee does not forage on *Arbutus unedo* because it lacks metabolic capabilities to use the main sterolic component from this plant, β-sitosterol [10]. However, this hypothesis has never been verified for wild species through experimental tests such as rearing.

In this paper, we performed a comprehensive nutrional study of five pollen diets by analysing their chemical contents (e.g. polypeptides, amino acids and sterols) and measuring their impact on the development and feeding behaviour of bumblebee micro-colonies. We addressed two specific questions: (i) what is chemical composition of a beneficial diet for the polylectic species *Bombus terrestris*, and (ii) do *Bombus terrestris* workers adapt their collection of pollen and/or nectar according to pollen quality?

## Materials and Methods

### Bumblebee model

We selected *Bombus terrestris* L. (Hymenoptera, Apidae) as a model of polylectic bee species. This social species forages on hundreds of different plant species and numerous plant families [20], [36], [37]. Nutritive value of pollen diet has even more impact on bumblebee development, as individual workers do not change the composition of the diet they supply to the brood, unlike honeybee [38]. It is a widespread and common bumblebee species throughout Europe and its rearing is quite easy and well-documented [17], [19], [39].

### Diets

We selected five pollen diets that are expected to have different effects on the development of a bumblebee colony. Based on previous study, Calluna vulgaris* (Ericaceae) and *Cistus* sp.* (Cistaceae) are considered as poor pollen resources for *Bombus terrestris* because they display low protein content and low performance for colony development [19]. Conversely *Cytisus scoparius* (Fabaceae), *Sorbus aucuparia* (Rosaceae) and *Salix caprea* (Salicaceae) are considered as good pollen diets [18], [19], [40]. These five pollen diets were prepared using honeybee pollen loads. Pollen loads of *Cytisus scoparius*, *Salix caprea* and *Sorbus aucuparia* were supplied by hives with pollen trap in particular areas and periods where the target plant is dominant. Commercial pollen samples were purchased from the company “Pollenergie France” for *Calluna vulgaris* and *Cistus* diets. This company sorts the incoming batches with high proportion of a dominant pollen species and stored them at −30°C for human consumption. We obtained five monofloral pollen diets by removing all non-target pollen based on the color of pollen loads. Purity of floral composition was checked under light microscope (LEITZ) at magnification of 400X or 1000X and compared with a reference pollen collection. A fraction of each monofloral diet was lyophilized and stored at −20°C for chemical analyses. The five monofloral pollen loads were mixed with inverted sugar syrup (BIOGLUC®, Biobest) (90% and 10% w/w respectively), which resulted in monofloral pollen pastes at 77% pollen dry matter. These pollen pastes were used to form monofloral candies, which were provided to the micro-colonies. Candies were weighted and stored at −20°C.

The different samples of pollen investigated did not involve endangered or protected species. No specific permits were required for the described field studies as pollen collection did not occur in privately owned or protected locations.

### Chemical analyses of pollen diets

#### Polypeptide analysis

Polypeptide content was assessed using five milligrams (dried weight) of each of five pollen diets in triplicate following the method described in [28]. The pollen was first ground by bead beating under nitrogen. The multi-step procedure can be summarized as follows: (i) three successive washes with TCA/acetone, methanolic ammonium acetate and acetone, respectively, to remove contaminants, (ii) elimination of acetone to pellet dryness, (iii) polypeptide extraction with a phenol/SDS mixture, (iv) polypeptide precipitation from phenol phase with methanolic ammonium acetate, (v) washes (methanol and acetone), followed by air-drying the polypeptide pellet, and (v) resuspension of the polypeptide pellet in a 4M guanidine HCl buffer.

Quantifications of total polypeptide content were performed using BCA Protein Assay Kit (Pierce, Thermo Scientific) with standard curve of BSA (bovine serum albumin).

#### Amino acid analysis

We added 1 ml of hydrolysis solution (6N HCl, 0.1% phenol and 500 µM norleucine) to 3–5 mg pollen (dried weight). The tube was put in liquid nitrogen for one minute to avoid methionine degradation (24 h, 110°C). The hydrolysate was dried by vacuum in a boiling bath at 100°C. Afterwards, 1 ml of buffer pH 2.2 was added into the tube. The sample solution was mixed and poured in a HPLC vial after filtration (0.2 µm). Total amino acids were measured separately with an ion exchange chromatograph (Biochrom 20 plus amino acid analyser).

Free amino acids were extracted from 30–50 mg (dry weight) pollen with 200 µl of extraction solution (1 mM norleucine, 0.1 N HCl and 2% thiodiglycol) in an ultrasonic bath for 30 min. Afterwards, 100 µl of 15% dihydrated 5-sulfosalicylic acid were added for precipitation of proteins in the ultrasonic bath for 5 min. After centrifugation (11 000 rpm for 5 min), 250 µl of the supernatant was poured into a microcentrifuge tube with filter (0.2 µm). After centrifugation (10 000 rpm) and membrane filtration for 10 min, 100 µl of a pH adjustment solution (1∶1, 1.5 N NaOH and pH 2.2 buffer) were added to 240 µl of the supernatant. The microcentrifuge tube was mixed and 200 µl of the sample were transferred into an insert for HPLC vial before measurement in the amino acid analyser.

For both extractions, norleucine constituted the internal standard allowing further amino acid quantification. Only tryptophan was omitted because its isolation requires a separate alkaline hydrolysis from additional amount of sample. Moreover, tryptophan is hardly ever a limiting essential amino acid [41].

#### Sterol analysis

Sterol content was analysed using 20 milligram samples of pollen (dried weight) according to the method described in [42]. The multi-step procedure can be summarized as follows: (i) saponification of the samples with methanolic potassium hydroxide, (ii) extraction of the unsaponifiable (USM) fraction with diethylether and several water-washings of the organic phase, (iii) evaporation of solvent, (iv) USM fractionation into its components using thin-layer chromatography (TLC), (v) derivatization of the sterols (scraped from the silicagel) into trimethylsilyl ethers (TMS), and (vi) separation of TMS by Gas Liquid Chromatography (GLC). The total sterol content was determined considering all peaks of sterols (at concentrations above the LOQ) eluted between cholesterol and betulin. Individual sterols – quantified on the basis of peak areas from analyses – were expressed as percentages of the total sterol content. Compounds were identified by comparing the relative retention times (β-sitosterol –TMS  = 1.00) with those of oil reference (sunflower oil with well-known composition). These identifications were checked by GC/MS (Gas Chromatograph/Mass Spectrometer) analyses [42].

### Micro-colony rearing

Two-day-old workers of *Bombus terrestris* were provided by Biobest *bvba* (Westerlo, Belgium). They were divided into 35 micro-colonies (seven micro-colonies for each diet) of four workers and placed in different plastic boxes (10×16×16 cm). These micro-colonies were reared in a dark room at 26–28°C and 65% relative humidity. They were fed *ad libitum* with inverted sugar syrup (BIOGLUC®, Biobest) and pollen candies during a 12-day period following the first episode of egg-laying of a worker.

To evaluate diet performance and bumblebee feeding response, we measured several parameters for each micro-colony (parameters adapted from [19]): (i) Number and fresh weight of larvae (total and mean weight of larvae), (ii) pollen collection (i.e. amount of pollen consumed and stored) per micro-colony (fresh matter), (iii) pollen efficacy which was calculated by dividing the total weight of larvae by the weight of pollen collected and (iv) syrup collection (i.e. amount of syrup consumed and stored). Pollen candies were weighed before introduction into the micro-colony (0.5 g, 1 g or 1.5 g depending on the age of the micro-colony) and again after their removing to measure pollen collection. New pollen candies were provided every two days.

Such a method using queenless *Bombus terrestris* micro-colonies for testing the nutritive value of pollen diets has been shown to be a good estimate of queenright colony development at least under laboratory conditions with *ad libitum* food [43].

### Data analysis

#### Univariate analyses

We performed a one-way analysis of variance (one-way ANOVA) to test the null-hypothesis of no-difference in quality criteria (see above) and in essential amino acid content between the pollen species. Since it is a parametric test based on an *F-*distribution, the following assumptions were checked: (i) independent observations, (ii) normality of the residuals (normal QQ-plot and Shapiro test) and (iii) homoscedasticity (Bartlett test). As all these assumptions were met (*p*-values>0.05 for Shapiro and Bartlett tests), the data were not transformed and the one-way ANOVA produced the *p-*value for each hypothesis test. When the ANOVA was significant, we performed multiple pairwise comparisons (post-hoc test). *P*-values were adjusted using Bonferroni's correction to avoid increases in type error I due to multiple testing. All data visualization and analyses were performed in R version 2.2.1 with Sciviews R Console (version 0.9.2) [44].

#### Multivariate analyses

In order to detect differences between the diet compositions (sterols and amino acids), we performed a perMANOVA using Bray-Curtis distances and 999 permutations (“adonis” command, R-package vegan, [45]). Prior to this permutational analysis of variance, the multivariate homogeneity of within-group covariance matrices was verified using the “betadisper” function implementing Marti Anderson's testing method. When the returned *p*-value was significant (*p*<0.05), multiple pairwise comparisons were conducted on the data; *p*-values were adjusted using Bonferroni's correction to avoid increases in type error I due to multiple testing. The differences were visually assessed on a non-metric multidimensional scaling (nMDS) ordination using a Bray-Curtis similarity matrix, two dimensions and 50 runs. Statistics were conducted in R using functions from ecodist [46], ellipse [47] and BiodiversityR [48]. Indicator compound analyses were also performed in R using the “indval” function from the labdsv package [49] to identify the compounds that were indicative of one diet. All multivariate analyses were conducted in R version 2.9.1 [50] using data expressed as concentrations in mg/g for each sterolic compound or amino acid (absolute abundances). In addition, the Bray-Curtis dissimilarity index was used to measure the deviation of essential pollen amino acid composition from the ideal composition determined for the honeybee by De Groot [16]; namely arginine 11%, histidine 5%, isoleucine 14%, leucine 16%, lysine 11%, methionine 5%, phenylalanine 9%, threonine 11%, tryptophan 4% and valine 14%. This Bray-Curtis index is often used by ecologists to determine dissimilarities between samples. Such use (i.e. for deviation from ideal composition) has been already made in similar study [28]. The Bray-Curtis index was calculated by R software as during perMANOVA analyses.

## Results

### Pollen nutritional contents

#### Polypeptide and amino acid contents

**Figure 1:**
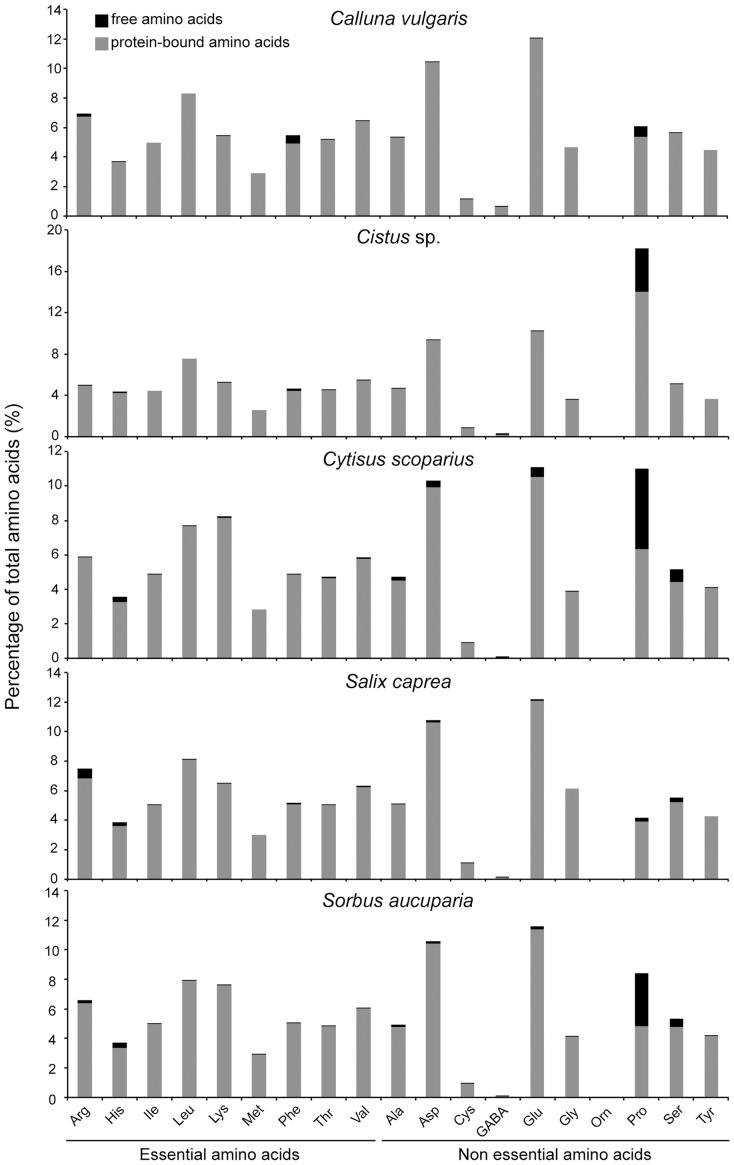
Amino acid profile of the five diets: *Calluna vulgaris*, *Cistus* sp., *Cytisus scoparius*, *Salix caprea* and *Sorbus aucuparia*. All measured amino acids are displayed and separated into free and protein-bound fractions. (Arg =  arginine, GABA =  γ-aminobutyric acid, His =  histidine, Ile =  isoleucine, Leu =  leucine, Lys =  lysine, Met =  methionine, Orn =  ornithine, Phe =  phenylalanine, Thr =  threonine, Val =  valine, Ala =  alanine, Asp =  aspartic acid, Cys =  cysteine, Glu =  glutamic acid, Gly =  Glycin, Pro =  proline, Ser =  serine, Tyr =  tyrosine).

Polypeptide contents of *C. scoparius*, *S. caprea* and *S. aucuparia* were quite similar, around 7–8% of lyophilized weight, but were lower for *Cistus* sp. and *C. vulgaris*, around 2% of lyophilized weight. These results were corroborated by the total amino acid contents, which were higher in *C. scoparius*, *S. caprea* and *S. aucuparia* (from 19% to 30% of lyophilized weight) than in *Cistus* sp. and *C. vulgaris*, around 14% of lyophilized weight (Table 1). Although the proportions of free and protein-bound amino acids were variable among the different diets, the proportion of essential amino acids was highly conserved in the different amino acid profiles, around 50% of total amino acids (Figure 1 and Table 2).

**Table 1:** Polypeptide and total amino acid contents from the five pure pollen diets expressed as percentage of lyophilized matter (mean ± sd).

Family – species	Polypeptide content (%)	Total amino acid content (%)
Cistaceae – *Cistus* sp.	1.94±0.20	13.53±0.59
Ericaceae – *Calluna vulgaris*	2.24±0.38	13.53±0.86
Fabaceae – *Cytisus scoparius*	7.73±0.66	30.07±2.34
Rosaceae – *Sorbus aucuparia*	7.38±0.42	23.55±0.47
Salicaceae – *Salix caprea*	7.15±0.84	18.65±0.89

**Table 2:** The total concentration of free and protein-bound amino acids as well as percentage of essential amino acids and deviation of ideal essential pollen amino acid composition for the five pollen diets (mean).

Pollen diet	Protein-bound AA (mg/g lyophilized weight)	Essential protein-bound AA (%)	Free AA (mg/g lyophilized weight)	Essential free AA (%)	De Groot deviation (Bray-Curtis index)*
*Calluna vulgaris*	132.98	49.43	2.33	45.90	0.072 d
*Cistus* sp.	128.47	45.68	6.84	10.50	0.091 bc
*Cytisus scoparius*	278.33	51.77	22.41	9.51	0.113 a
*Salix caprea*	182,49	51.39	4.02	57.12	0.097 b
*Sorbus aucuparia*	222.06	49.43	13.5	13.73	0.086 c

AA =  amino acids.

Numbers (mean) with the same letter are not significantly different.

Although the five diets contained the full spectrum of essential amino acids, one-way ANOVA showed significant difference between the five pollen diets according to their essential amino acid content (*F*_4,11_ = 29258, *p*<0.001). *C. scoparius*, *S. caprea* and *S. aucuparia* displayed the highest concentrations of essential amino acids with, on average, 146 mg/g, 96 mg/g and 112 mg/g of lyophilized pollen, respectively. However *C. scoparius* had a significantly less ideal composition of essential pollen amino acids on the basis determined by [16] for honeybees than the other four plants investigated (*F*_4,11_ = 33.312, *p*<0.001, Table 2). Nevertheless Bray-Curtis dissimilarity index between pollen diet and the ideal composition remained low (Table 2). Only isoleucine and to a lesser extent valine were found in smaller proportions than determined by De Groot [16].

**Figure 2:**
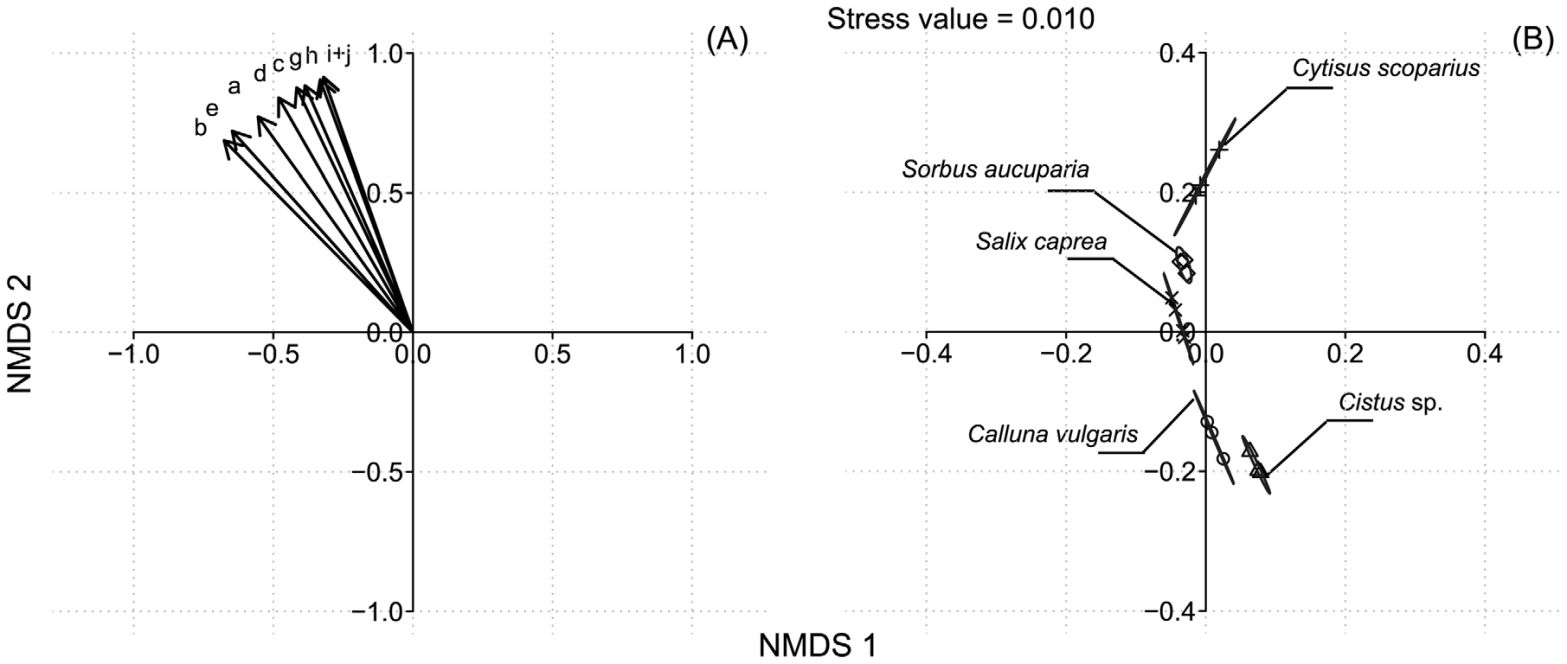
nMDS ordination plot based on Bray-Curtis distances calculated on absolute amounts (mg/g) of essential amino acids in pollen diets showing (A) essential amino acids vectors a, arginine; b, histidine; c, isoleucine; d, leucine; e, lysine; f, methionine; g, phenylalanine; h, threonine and i, valine; and (B) replicates within pollen type (n = 3, except for *Salix caprea*, n = 4). Stress value  = 0.010.

PerMANOVA detected a significant difference in the composition of essential amino acids (free and protein-bound pooled) among the pollen diets (*F*_4,11_ = 124.02, *p*<0.001). Pairwise comparisons arranged the different pollen species into three groups: (i) one with *C*. *scoparius, S. aucuparia* and *Cistus* sp., (ii) one with *S. caprea*, and (iii) one with *C. vulgaris* which did not significantly differ from *Cistus* sp. Indicator Compound Analysis showed that all essential amino acids were significantly associated with *C. scoparius* pollen because of their higher concentrations in this diet. The decrease in concentration of essential amino acids among the pollen diets was well reflected by the gradient along NMDS 2 on nMDS ordination (stress value  = 0.010, Figure 2).

#### Sterol content

The different pollen diets displayed concentrations of total sterols from 2.5 to 9.6 mg/g of lyophilized matter, for *Cistus* sp./*Cytisus scoparius* and *Sorbus aucuparia*, respectively (Table 3). The major phytosterols were common C_29_ sterols like β-sitosterol and δ5-avenasterol but in some pollen, we found also high amounts of δ7-avenasterol (*C. vulgaris*, 20.23%) or 24-methylenecholesterol/campesterol fraction (*S. aucuparia*, 84.07%). In all pollen diets, cholesterol, desmosterol, stigmasterol, cholestenone, δ7-avenasterol (except for C. *vulgaris*) and δ7-stigmasterol concentrations were low (Table 3).

**Table 3:** Sterolic compounds from the five pure pollen diets.

Sterols	*Calluna vulgaris* (n = 5)	*Cistus* sp. (n = 2)	*Cytisus scoparius* (n = 3)	*Salix caprea* (n = 3)	*Sorbus aucuparia* (n = 4)
Cholesterol	4.01±1.38	0.24	6.22±2.09	4.37±2.40	2.03±1.27
Desmosterol	0.09±0.10	0.87	<LOD	0.16±0.10	0.24±0.21
24-Methylenechol./campesterol^a^	6.28±2.23	**22.21**	**20.08±3.01**	**7.20±3.44**	**84.07±1.13**
Stigmasterol	0.57±0.19	1.11	0.44±0.38	0.47±0.17	0.72±0.11
Unk. 484	<LOD	12.88	<LOD	<LOD	<LOD
β-sitosterol	**25.96±2.89**	**12.02**	**51.24±2.16**	**45.82±6.32**	**3.00±0.51**
δ5-avenasterol	**37.86±3.07**	**42.58**	**20.36±4.2**	**38.45±4.37**	**9.15±1.45**
Cholestenone	0.64±0.61	1.94	0.14±0.13	0.34±0.59	0.07±0.14
δ7-stigmasterol	4.36±0.31	3.68	0.5±0.4	1.69±0.39	0.33±0.36
δ7-avenasterol	**20.23±4.21**	2.48	1.02±0.9	1.51±1.49	0.40±0.50
TOTAL (mg/g in lyophilized matter)	7.36±2.17	2.47	2.46±1,10	5.33±1.05	9.64±1.68

The concentrations are expressed as percentage of total sterolic compounds. The three most abundant sterols in the investigated samples are printed in bold. < LOD, under limit of detection.

^a^ Under the analytical conditions applied campesterol and 24-methylenecholesterol are nearly impossible to separate; the results are therefore pooled.

**Figure 3:**
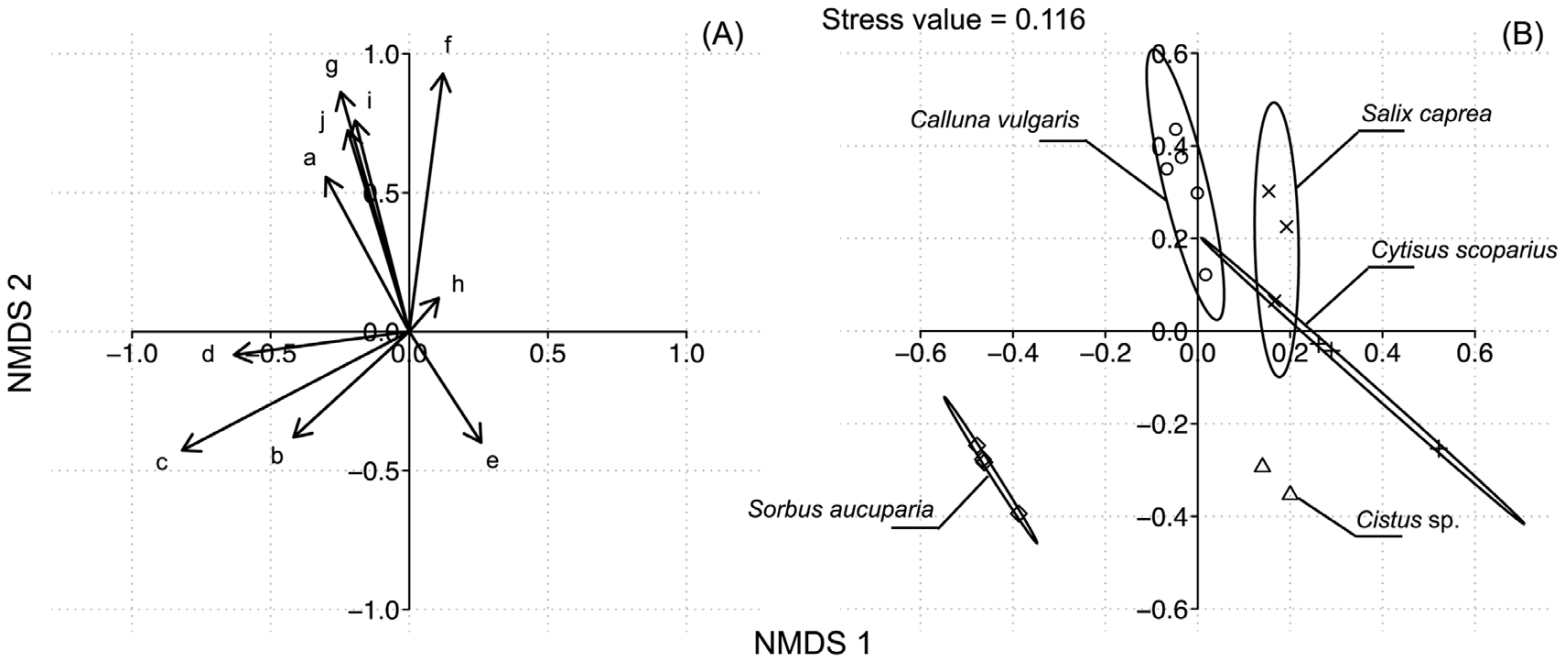
nMDS ordination plot based on Bray-Curtis distances calculated on absolute amounts (mg/g) of sterolic compounds in pollen diets showing (A) sterolic vectors with a, cholesterol; b, desmosterol; c, 24-methylenecholesterol/campesterol; d, stigmasterol; e, unk.484; f, β-sitosterol; g, δ5-avenasterol; h, cholestenone; i, δ7-stigmasterol and j, δ7-avenasterol; and (B) replicates within pollen type (n are mentioned in **Table 3**). Stress value  = 0.116.

PerMANOVA detected a significant difference in sterolic composition between the pollen diets (*F*_4,12_ = 23.49, *p*<0.001). Pairwise comparisons and nMDS ordination arranged the different pollen species into three distinctive groups: (i) one with C. *vulgaris*, (ii) one with *S. caprea*, *C. scoparius* and *Cistus* sp. and (iii) one with *S. aucuparia* which did not significantly differ from *Cistus* sp. (*F*_1,4_ = 54.77, *p* = 0.058) (stress value  = 0.116; Figure 3). Indicator Compound Analysis showed that 24-methylenecholesterol/campesterol fraction was significantly associated with *S. aucuparia* pollen (*p* = 0.024, indicator value  = 82.26%), and δ7-avenasterol (*p* = 0.010, indicator value  = 0.896) as well as δ7-stigmasterol (*p* = 0.018, indicator value  = 0.626) with *C. vulgaris.*

### Micro-colonies development

One-way ANOVA detected a significant difference between the five pollen diets (*F*_4,27_ = 12.87, *p*<0.001) with regard to total weight of larvae. Post-hoc testing arranged the different pollen species into three distinctive groups: one for the *C. scoparius, S. aucuparia* and *S. caprea*, one for *C. vulgaris*; and one for the *Cistus* sp. As no significant difference was detected in number of larvae among the treatments (*F*_4,27_ = 1.66, *p* = 0.189), the analyses clearly showed that pollen diet significantly influenced the mean weight of larvae (*F*_4,27_ = 6.87, *p*<0.001): micro-colonies raised with *C. vulgaris, C. scoparius, S. aucuparia* or *S. caprea* pollen diet produced significantly larger larvae (from 0.29 g to 0.36 g) than those raised with pollen of *Cistus* sp. (0.12 g) (Table 4).

**Table 4:** Parameters of pollen quality measured over a 12-day period for each diet.

Criteria of pollen quality	*Calluna vulgaris*	*Cistus* sp.	*Cytisus scoparius*	*Salix caprea*	*Sorbus aucuparia*
Total weight of larvae (g) ^(1)^	2.52±0.60	0.93±0.65	3.77±0.82	2.78±0.99	3.05±0.68
Mean weight of larvae (g)	0.29±0.11	0.12±0.04	0.31±0.10	0.29±0.08	0.36±0.12
Number of larvae	10±4	7±4	13±4	11±5	9±4
Weight of collected pollen (g) ^(2)^	3.90±0.63	3.84±0.61	3.27±0.40	3.26±0.89	3.08±0.34
Pollen efficacy ^((1)/(2))^	0.65±0.11	0.24±0.18	1.15±0.13	0.77±0.22	0.98±0.14
Syrup collection (ml)	63.86±3.67	65.14±2.27	53.67±7.20	59.43±5.38	60.57±4.54

The mentioned values are the mean ± sd for seven bumblebee micro-colonies.

**Figure 4:**
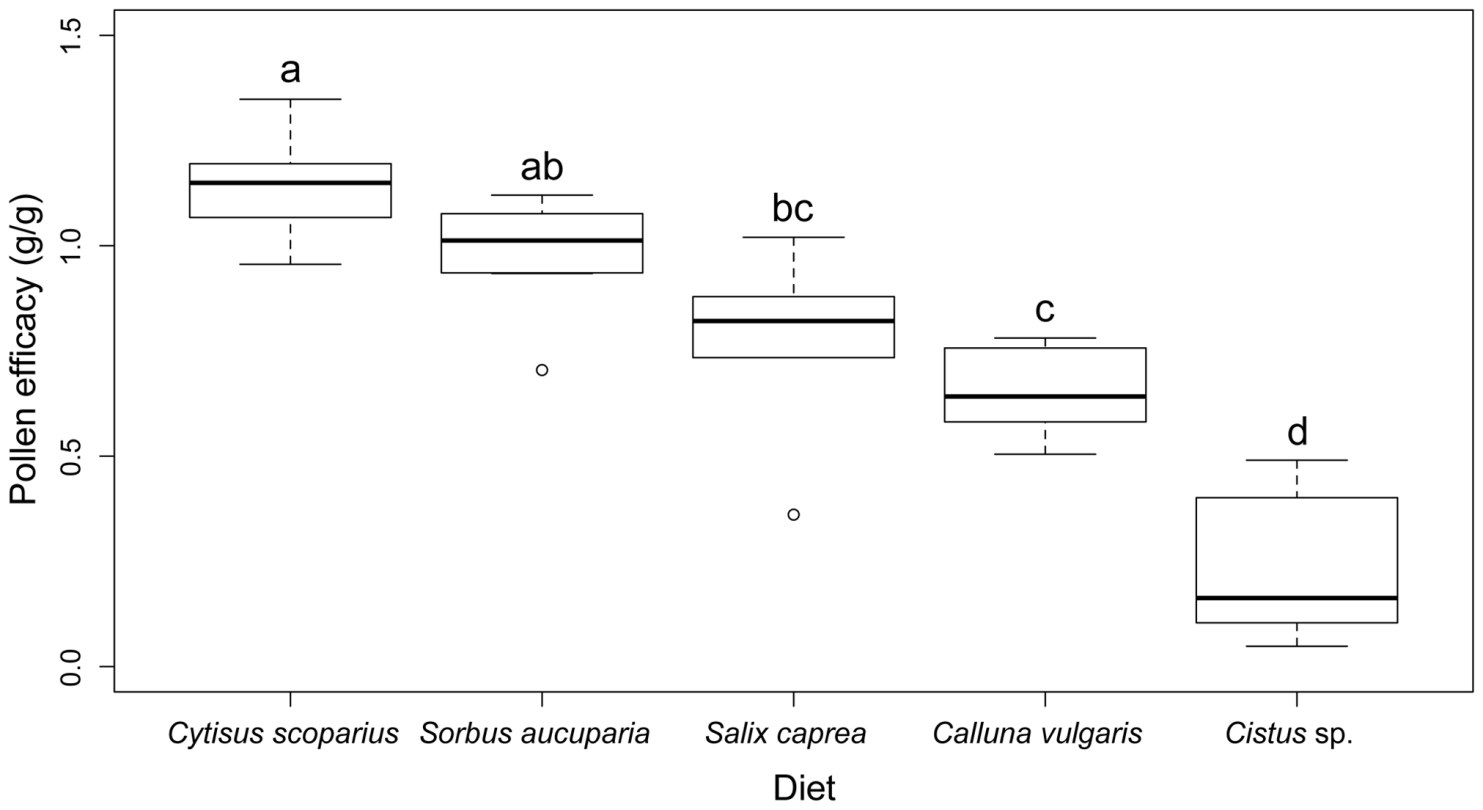
Pollen efficacy for micro-colonies fed on different diets over a 12-day period. Differences across diets were significant (ANOVA: *p*<0.001, *F* = 30.05; see Results). Groups differing significantly from each other in posthoc test are marked with different letters above box-plots, shared letters indicating non-significant differences. Medians are given in box-plots, and an outlier is marked with a circle.

One-way ANOVA did not show significant differences in pollen consumption (total weight of consumed pollen) (*F*_4,29_ = 2.59, *p* = 0.057) but analyses of pollen efficacy showed significant differences (*F*_4,27_  = 30.05, *p*<0.001). Side-by-side box plot diagram and post hoc tests separated the five pollen diets into four groups (Figure 4). Efficacies of diet from the pollen of *C. scoparius* (1.15 efficacy) and *S. aucuparia* (0.98 efficacy) were significantly higher than those constituted with pollen of *C. vulgaris* (0.65 efficacy) or *Cistus* sp. (0.24 efficacy). *Salix caprea* diet offered intermediate results with 0.77 efficacy.

Interestingly, the pollen diet provided to the micro-colonies appeared to affect their consumption of syrup (ANOVA, *F*_4,29_ = 5.60, *p* = 0.002) (Table 4). Pairwise comparisons detected significant differences between *C. scoparius* and *Cistus* sp. (*t* = −4.30, *p* = 0.002) as well as between *C. scoparius* and *Calluna vulgaris* (*t* = −3.82, *p* = 0.005).

## Discussion

### Chemical composition of a beneficial diet for bumblebees

Our results show that *ad libitum* pollen collection per micro-colony of *Bombus terrestris* is independent of pollen chemical composition. Workers do not adjust their own consumption and larval provisions to compensate for low nutritional quality of pollen diet. Consequently larval mean weight (i.e. micro-colony development) differs significantly according to pollen diet. Variability in pollen efficacy is therefore directly dependent on pollen nutritional quality and not on other factors like differences in larval feeding stimuli [51] or presence of chemical repellent [52]. These findings are congruent with similar studies, which have highlighted *Bombus terrestris* feed on similar amount of pollen despite the protein quality of pollen [18], [19].

As the diets investigated were similar in their composition of amino acid (i.e. similar proportions of aspartic and glutamic acids important for nitrogen assimilation as well as full spectrum of essential amino acids), this nutritional trait is probably not responsible for the observed reduced performance of *Calluna* and *Cistus* diets. These results are consistent with previous studies suggesting that amino acid profiles are highly conserved among plants [22], [27]. However investigated diets differ strongly in their proportions of free and protein-bound amino acids. Among the five pollen species, *Cytisus scoparius* displays the highest percentage of free amino acids (7%) and was the diet that led to the highest mortality rate during our three weeks experiment (unpubl. data). Direct toxic effects of non-protein amino acids have been already highlighted in previous studies and occur through several mechanisms, including misincorporation into proteins, obstruction of primary metabolism, and mimicking and interfering with insect neurological processes [53].

A significant association was found between polypeptide/amino acid concentrations of pollen and pollen efficacy. Previous studies have already documented that rich protein resources seem to be linked to efficient development of bumblebee colonies [17]–[19] and floral preferences [11].

When we compared the phytosterols composition with pollen efficacy, 24-methylenecholesterol, β-sitosterol and δ5-avenasterol appear to be positively associated to bumblebee larval development. It is now well established that 24-methylenecholesterol is the precursor of Makisterone A (i.e. 24-methyl-20-hydroxyecdysone), a 28-carbon ecdysteroid which has been isolated from the ovaries of *Apis mellifera* [54], [55]. 24-methylcholesterol is therefore an essential sterol in bee metabolism influencing moulting and the development of ovaries [33], [56], [57]. Whereas β-sitosterol is known to have antifeedant effects on *A. mellifera*, pollen rich in β-sitosterol (e.g. *Arbutus unedo*) is freely collected by *B. terrestris* [10]. This phytosterol as well as δ5-avenasterol might be involved in some metabolic pathways of *B. terrestris* or have a phagostimulant effect on this bumblebee species [58]. Moreover phytosterols were described as stimuli of foraging behaviour in *Apis mellifera* [59], [60].

Based on the present study, high concentration of polypeptides/amino acids, low concentration of free amino acids and abundance of 24-methylenecholesterol, β-sitosterol or δ5-avenasterol appear to promote production of larger larvae in *B. terrestris* micro-colonies. Larger workers can be better at foraging than smaller workers, bringing back more nectar per unit time, removing more pollen from buzz-pollinated flowers, flying at cooler temperatures, probing deeper flowers, and possibly being less prone to predation [61]–[66]. Worker size also positively correlates with the numbers of egg cells and emerging workers produced [67], while larger queens have greater hibernation survival and reproductive success [68], [69]. Previous work has found that micro-colonies can be a reasonable analogue for whole colonies when testing the effects of pollen diets, at least under laboratory conditions with *ad libitum* food [43], so our results suggest pollen diet may impact offspring size and then potentially fitness in *B. terrestris* colonies. However, experiments with full-size colonies in the field will be necessarily to validate this, particularly in the context of natural variation in pollen diet composition.

### Selection of pollen chemical compounds in floral reward

Previous studies demonstrated that bumblebees could select pollen of high chemical quality (i.e. with high protein concentration; other compounds like sterols were not tested) [11], [20], [70], [71], possibly based on perception of particular volatile compounds [72]. Moreover bumblebees appear quite flexible in their host plant use as they can readily switch from one plant species to another that was previously less preferred but can display most abundant rewards at a given period [37]. Our results additionally show that workers could not change their pollen collection as a function of pollen quality. This kind of feeding behaviour (i.e. constant amount of collected pollen) was already described in the honeybees and the generalist, primitively eusocial bee *Lasioglossum zephyrum* [22], [60]. However, bumblebees might offset poorer nutritive value with larger syrup consumption (i.e. nectar foraging) to improve the cost-benefit balance of foraging activity.

Pollen of poor quality has a negative impact on offspring size and probably leads to high energetic cost related to additional nectar foraging. This fundamental aspect probably leads to the selection of foragers able to detect rewards of good chemical quality [70]. Preferential selection of pollen may be one factor by which bees influence evolutionary modification in floral traits [73], [74]. Conversely, floral rewards of high quality in polypeptides, amino acids and sterols could be an adaptive response of plants to attract bumblebees and to promote their fidelity. Growing evidence suggests that chemical traits of pollen could be considered as a pollination syndrome since they can shape bee-flower interactions [11].
